# Perception among Healthcare Professionals of the Use of Social Media in Translating Research Evidence into Clinical Practice in Mangalore

**DOI:** 10.1155/2018/7573614

**Published:** 2018-11-21

**Authors:** Bhaskaran Unnikrishnan, Priya Rathi, Daivik Shah, Abhay Tyagi, Anish V. Rao, Koyel Paul, Joe Tomy

**Affiliations:** ^1^Department of Community Medicine, Kasturba Medical College, Mangalore, Manipal Academy of Higher Education, Manipal, Karnataka, India; ^2^Kasturba Medical College, Mangalore, Manipal Academy of Higher Education, Manipal, Karnataka, India

## Abstract

**Introduction:**

Social media has a potential to bring about major changes in the healthcare system.

**Objective:**

To find out the pattern of use of social media among healthcare professionals (HCPs) and perception, facilitators, and barriers of using social media, to translate evidence into clinical practice.

**Method:**

We conducted a cross-sectional study among 196 HCPs of institutions attached to a university using a self-administered questionnaire.

**Result:**

97.3% used social media; however, only 63.4% used it for research. YouTube was the most preferred media. Majority of people believed that social media enables wide range of evidence over the shorter span of time, poses a threat to privacy, and cannot replace face to face interaction. Perceived barriers were the privacy concern, unprofessional behavior, lack of reliability, and information overload.

**Conclusion:**

There is a need for the development of appropriate guidelines for sharing the research output among various stakeholders using social media.

## 1. Introduction

Translation of research is a dynamic and interactive process that includes the synthesis, dissemination, exchange, and ethically sound application of knowledge to improve health, provide more effective services and products, and strengthen the healthcare system [1].

From the year 2006, there has been tremendous increase in research output from India; however, the accessibility to all stakeholders comes with a cost either in the form of subscriptions cost or article processing charges to authors for open access articles. Indians spend close to $2.4 million annually to get their scientific research output published in different open access (OA) journals [2]. Many Indian institutions have subscription to publishers, and due to higher Gross Domestic Product (GDP) as compared to other developing countries, paid international journals are not freely available to all. It is known that 86% of research evidence is not converted to clinical practice, as it fails to reach the considerable number of population of professionals in the shorter time span. This has created a huge gap in research output and its accessibility to many stakeholders [2–4].

There has been a substantial growth in the use of social media (SM) within healthcare [4].

SM enables the user to establish digital communication with another user, a two-way discussion, provide feedback, and share information between stakeholders [5]. It has been documented that usage of SM increases the number of reads or download of an article. Allen et al. found a moderate correlation between tweets and number of citations [6]. Another study showed an eleven-time likelihood of increase in the number of reads for tweeted OA articles.[7] Wang et al. (2015) showed that OA articles get more attention in SM. Many prestigious journals and publishers have their own dedicated social media tool, which increases the impact of published articles [8].

There are many documented hindrances of usage like lack of time, skills, unawareness, and ethical issues [9]. Hence, we planned to study the pattern, perception, barriers, and facilitators of using SM in research translation in our healthcare setting.

## 2. Materials and Methods

We conducted a cross-sectional study in healthcare institutions and hospitals in Coastal Karnataka. This included one medical, one dental, and one allied health institution affiliated to a deemed to be a university in India. The university promotes research activities and facilitates research in all disciplines. Our study participants were HCPs inclusive of postgraduates in medical, dental, and allied health sciences and in preclinical, para-clinical, and clinical specialties in medical, dental, and faculty members of allied health science. Current undergraduates and interns were excluded as research is not a part of undergraduate medical education curriculum in India. The sample size was calculated based on assumption that 15% of HCPs used SM to translate research evidence into clinical practice as per the previous study [1]. Taking 5% as absolute precision and 95% as confidence interval with a power of 80%, sample size came to be 196 and sampling was done using convenience sampling method. After obtaining IEC approval and permission from heads of institutions, we approached study participants and explained the purpose of our study to them, and a written informed consent was taken. Those who consented for the study were given a self-administered questionnaire to fill in on their own. It was a semistructured questionnaire which was content validated by two experts. Review on various components of questionnaire for accuracy and relevance of each item was focused on. In case of discrepancy, a third expert's opinion was taken into consideration. The questionnaire was pilot-tested among 30 participants to check for reliability; a Cronbach's alpha of 0.853 was obtained. Following are the components of the questionnaire (appendix): sociodemographic characteristics, usage of SM for research translation, perception, facilitators, and barriers of using SM to translate evidence into practice. The filled questionnaire was collected back on a later date. Those participants who did not fill the questionnaire even after paying two visits to them in their workplace within a gap of one week were not included in analysis.

The data collected were entered into MS Excel and analysis was done using Statistical Package for Social Sciences Services version 11.5 and it is summarized using median (IQR) and proportions.

## 3. Results

We approached 196 HCPs out of which 183 were willing to take part in our study, resulting in a response rate of 93.3%. The mean age was found to be 33.6 (9.75) years and most of the participants were in the age group of 36-45 years. Among the participants, 55.2% were female, 66.7% were in the medical profession, 77% were postgraduates, and 51.9% had less than 5 years of experience in their work. Most of the participants were involved in research (89.1%), 36.6% had their first exposure to research in their postgraduation dissertation, and 62.3% of participants had less than 5 years of experience in research (Table 1). Almost all of the participants (97.3%) used SM for general purpose and 63.4% used it for research translation. YouTube was the most preferred media (38.8%), followed by WhatsApp (38.3%) (Table 2). In the study, 43.2% of participants used SM to obtain the research evidence while only 17.5% used SM to disseminate research evidence as depicted in Table 3. Majority of people believed that SM enables availability of wide range of evidence over the shorter span of time; SM poses a threat to privacy and copyright issues. They also believed that SM usage is not age bound and not unprofessional (Table 4). Perceived barriers for using SM for research translation were privacy concern, concerns of unprofessional behavior, lack of reliability, and information overload, whereas easy accessibility, low cost, no training needed, open access to free available resource, and availability of professional forum was perceived facilitators for the use of SM in evidence generation and dissemination (Table 5).

## 4. Discussion

SM can aid in translation of evidence into practice in following ways: platform for sharing information, discussion of new healthcare policies, behavior change for health promotion, community participation, and patient interaction [9–12]. HCPs can use SM to potentially improve health outcomes, develop a professional network, increase personal awareness of scientific news and discoveries, motivate patients, and provide health information to the community [10, 13].

Our study highlighted that two-thirds of HCPs used social media for research translation, with the most common purpose being gathering or obtaining evidence. This was similar to the findings of other studies [14–17]. In our setting, HCPs utilized multiple SM platforms for research. You Tube, WhatsApp, and Facebook were used more frequently, which was identical to general usage pattern in India [18]. Utilization of YouTube and Facebook has been explored in disseminating and gathering research evidence. Any evidence that requires skill development can be disseminated by videos in YouTube as it provides direct visualization of process [19–23].

Only few HCPs utilized SM for disseminating their research findings (19%) and professional networking (23%). This could be due to the fact that HCPs like other general population primarily utilize SM for entertainment purpose with lack of awareness/understanding regarding its scope and benefits in evidence translation.

HCPs agreed that SM platforms are easily accessible and affordable, do not require any training to use, provide wide range of content in shorter span of time, and facilitate professional forum for discussion. At the same time, they also agreed that the reliability with information overload, privacy, copyright issues, and concerns of unprofessional behavior were the hindering factors for the limited usage of SM for professional purposes. Similar studies found that lack of reliability becomes a major limitation to SM use for clinical practice [4, 24–27].

In our study, most of the participants felt that use of SM is not age bound. However, a survey conducted in the USA found that most of their participants who used SM were below 40 years of age [27]. Another study conducted in Australia, India, and Malaysia found that 65.7% of their participants using SM were below 34 years of age [4]. Comparing our data with other studies, we got a similar SM usage pattern in the age group between 30 and 45 years. With this data, we can conclude that not all age groups are using SM for research translation.

Participants also believed that no special training is required to use SM. However, several studies conducted found that basic training is needed to learn how to use SM and navigate SM technologies. A study conducted in Australia, India, and Malaysia found that over half of the participants (53.3%) felt need for training to use SM to translate research evidence into clinical practices which necessitates training for widespread use.

Guarded and judicious use of SM provides potentials platform which can be utilized in translating evidence into practice in health promotion, prevention, cure, and professional development. However, due to threats like privacy, copyright issues, and unethical behavior, it is still underutilized by HCPs in our institution. Appropriate guidelines to prevent and tackle such issues can be formulated for optimum utilization of such low cost, widely acceptable platform.

India spends only 1.2% GDP in healthcare, and there is also scarcity of human resources in healthcare [26–30]. Evidence of practice gaps still exits.[30] Factors that contribute to this problem include lapses in communication between researchers and practitioners. Presently SM has become a tool of choice for obtaining information, which is evident from our study too. If generated evidence is channeled using SM, it has potential to reach many stakeholders in no time and at no cost.

**Limitations:** Being a single centric, the study may have limited generalizability. However, it includes institutions actively involved in research and institution based guideline, which may optimize the usage of SM in translation of evidence into practice within the organization. A multicentric study involving centers in different parts of country and formulating guideline for SM usage is a way forward.

## Figures and Tables

**Table 1:** General information of study participants (n=183).

**Characteristics**	**n **(%)
**Age(years)**	
≤25	41(22.4)
26-35	75(41)
36-45	**46(25.1)**
≥46	21(11.5)

**Gender**	
Male	82(44.8)
Female	**101(55.2)**

**Highest education**	
Graduate	31(16.9)
Post-graduate	**141(77)**
Super Specialty	11(6)

**Profession**	
Medical	**122(66.7)**
Dental	41(22.4)
Allied Health Science	20(10.9)

**Years of experience**	
Less than 5	**95(51.9**)
5-10	37(20.2)
More than 10	51(27.9)

**Involvement in research**	
Yes	**163(89.1)**
No	20(10.9)

**First exposure to research**	
Individual research	45(24.6)
Funded project	11(6)
PG dissertation	**67(36.6)**
Student Research	37(20.2)
Others	3(1.6)

**Years of experience in research**	
Less than 5	**114(62.3)**
5-10	36(19.7)
More than 10	33(18)

**Table 2:** Social media usage pattern in research among study participants (n=183).

**Pattern of usage**	**n **(%)
Social media usage among study participants	**178(97.3)**

Social media usage among study participants for research translation	**116(63.4**)

**Social media platform used among study participants for research translation**	

**YouTube**	**71(38.8**)

**WhatsApp**	**70(38.3**)

Facebook	53(29)

LinkedIn	33(18.0)

Blogs	27(14.8)

Instagram	22(12)

Pinterest	19(10.4)

Quora	16(8.7)

Twitter	13(7.1)

Microblogs	10(5.5)

Hike messenger	6(3.3)

Digg	5(2.7)

Reddit	4(2.2)

Connotea	3(1.6)

Tumblr	3(1.6)

Snapchat	3(1.6)

Others	3(1.6)

**Table 3:** Purpose of using social media for research among study participants (n=183).

**Purposes**	**n **(%)
Obtaining research evidence	**79(43.2)**

To guide postgraduate research	67(36.6)

To obtain updates in research or new evidence in general	64(35)

Evidence based health intervention, health promotion and health education	60(32.8)

Professional networking for research evidence and translation	43(23.5)

Taking part in research based forum discussion on new evidence.	41(22.4)

Building evidence based awareness among target audience	36(19.7)

Disseminating information on evidence based health intervention, health promotion and health education	35(19.1)

Disseminating original articles	32(17.5)

Obtain and disseminate information on research oriented student exchange programs	30(16.4)

Collaborations for evidence generation and translation	26(14.2)

**Table 4:** Perception of social media use in translation of research evidence into clinical practice among study participants (n=183).

**Statements**	**Median(IQR)**
Use of social media is unprofessional	2(2,3)

Social media usage will require additional training which limits its usage	3(2,4)

Social media poses a threat to privacy and copyright issues	4(3,4)

Social media facilitates multicultural forum for discussion on research evidence	4(3,4)

Social media jargons are difficult to understand	3(2,4)

Social media usage is age bound	2(2,4)

Social media enables availability of wide range of evidence over a shorter span of time	4(4,4)

Social media use poses legal consequences	3(3,4)

Social media cannot replace face to face interaction	4(3,4)

1: strongly disagree; 2: disagree; 3: neutral; 4: agree; 5: strongly agree.

**Table 5:** Barriers and facilitators in social media use in translation of research evidence into clinical practices among study participants (n=184).

**Barriers**	**Median(IQR)**
Time consuming	3(2,4)

Privacy concerns	4(4,4)

Concerns of unprofessional behavior	4(3,4)

Cannot reach my target audience	3(3,4)

Lack of reliability	4(3,4)

Lack of research evidence availability	3(2,4)

Information overload	4(3,4)

Lack of training to use social media	3(2,4)

Impose threat to my career	3(2,3)

Lack of accessibility	3(2,4)

**Facilitators**	

No training needed	4(3,4)

Easily accessible	4(4,4)

Less cost required for usage	4(3,4)

Open access with free available resource	4(4,4)

Authenticated resource availability/acceptability	3(2,4)

Availability of a professional forum	4(3,4)

1: strongly disagree; 2: disagree; 3: neutral; 4: agree; 5: strongly agree.

## Data Availability

The quantitative data used to support the findings of this study are available from the corresponding author upon request.

## Appendix

*The Questionnaire*

*Title.* Perception and attitude among healthcare professionals of the use of social media in translation of research into clinical practice

*About the Questionnaire*

- This questionnaire should take approximately 15 minutes to complete.

**Thank you very much for your cooperation!**

### A. General Information

(1) S.no.

(2) Age:

(3) Gender: Male/Female

(4) Highest education: Graduate/Postgraduate/Super Specialty

(5) Profession: Medical/Dental/Allied Health Science

(6) Year of experience: ____________

(7) Have you ever been involved in any kind of scientific research? Yes/No

(8) Which was your first exposure to research: Individual research /funded project/PG dissertation/ Student research?

(9) Others (specify)_________________

(10) Years of experience in research_________

### B. Social Media Usage in Research

(11) Do you use social media? Yes/No

(12) Do you use social media for research translation? Yes/No

(13) Which of the following social media do you use for research translation (put a tick mark) (multiple options can be chosen)?

**Table:**

(1) Facebook	(9) Microblogs
(2) WhatsApp	(10) Hike messenger
(3) Twitter	(11) LinkedIn
(4) Instagram	(12) Digg
(5) Quora	(13) Pinterest
(6) you tube	(14) Connotea
(7) Blogs	(15) Reddit
(8) Tumblr	(16) Snapchat
	(17) Others (specify)______________________

### C. Purpose of Using Social Media in Research

(14) For what purposes do you use social media in research (multiple options can be chosen)?

**Table:**

**Statement**	(Put a tick mark)
Obtaining research evidence for own research	
To guide Postgraduate research	
To obtain updates in research or new evidence in general	
Evidence based health intervention, health promotion and health education	
Professional networking for research	
Taking part in research based forum discussion	
Building awareness among target audience	
Disseminating information on evidence based health intervention, health promotion and health education	
Disseminating original articles	
Obtain and disseminate information on research oriented student exchange programs	
Collaborations for evidence generation and translation	
Providing online consultations on research evidence generation and dissemination	
Advocacy	

### D. Perceptions of Social Media Use in Translation of Evidence into Clinical Practices

(15) What is your opinion on the following?

**Table:**

**Statements**	SA	A	N	D	SD
Use of social media is unprofessional					
Social media usage will require additional training which limits its usage					
Social media poses a threat to privacy and copyright issues					
Social media facilitates multicultural forum for discussion on research evidence					
Social media jargons are difficult to understand					
Social media usage is age bound					
Social media enables availability of wide range of evidence over a shorter span of time					
Social media use poses legal consequences					
Social media cannot replace face to face interaction					

SA: Strongly Agree; A: Agree; N: Neutral; D: Disagree; SD: Strongly Disagree

### E. Barriers and Facilitators in Social Media Use in Translation of Evidence into Clinical Practices

(16) Which of the following are the barriers in social media use in translation of evidence into clinical practices?

**Table:**

Statements	SA	A	N	D	SD
Time consuming					
Privacy concerns					
Concerns of unprofessional behavior					
Cannot reach my target audience					
Lack of reliability					
Lack of research evidence availability					
Information overload					
Lack of training to use social media					
Imposing threat to my career					
Lack of accessibility					
Environmental concern					
Fear of losing credibility					

SA: Strongly Agree; A: Agree; N: Neutral; D: Disagree; SD: Strongly Disagree

(17) Which of the following are the facilitators in social media use in translation of evidence into clinical practices?

**Table:**

Statements	SA	A	N	D	SD
No training needed					
Easily accessible					
Less cost required for usage					
Open access with free available resource					
Authenticated resource availability/acceptability					
Availability of a professional forum					

SA: Strongly Agree; A: Agree; N: Neutral; D: Disagree; SD: Strongly Disagree
